# The Combination of Thought-Stopping And Exposure and Response Prevention in the Treatment of Predominant Obsessions: A Case Report

**DOI:** 10.7759/cureus.29226

**Published:** 2022-09-16

**Authors:** Ankit Sinha, Subho Chakrabarti

**Affiliations:** 1 Psychiatry, All India Institute of Medical Sciences, Bhubaneswar, IND; 2 Psychiatry, Postgraduate Institute of Medical Education and Research, Chandigarh, IND

**Keywords:** religious obsessions, predominant obsessions, response prevention, exposure, thought-stopping

## Abstract

The behavioral technique of thought-stopping is no longer used to treat obsessive-compulsive disorder (OCD) because of its ineffectiveness and concerns about the detrimental effects of thought suppression. However, it can be effective when used as a part of exposure and response prevention (ERP) treatment in those with predominant obsessions without overt compulsions. We present the case of a female with long-standing medication-resistant obsessions without compulsions. The combination of thought-stopping, ERP, and simple techniques to address neutralization and dysfunctional cognitions was effective in reducing her symptoms. Treatment gains were maintained for two years. The successful treatment of this patient with a combination of thought-stopping with ERP suggests that it might be worthwhile to examine the effectiveness of this integrated treatment in properly controlled trials of patients with predominant obsessions.

## Introduction

The behavioral technique of thought-stopping has been used to treat distressing thoughts and ruminations in different psychiatric conditions, especially obsessive-compulsive disorder (OCD). This relatively simple procedure systematically teaches patients to deliberately block unpleasant obsessional thoughts on their own [1,2]. Despite considerable interest in this method from the 1970s to the 1990s, thought-stopping is no longer used as a treatment for OCD. The principal reason is its ineffectiveness [2]. Despite many positive reports of thought-stopping among patients with OCD, properly conducted randomized trials are scarce, and their results are disappointing [1-4]. An analysis of four trials of thought-stopping among 50 patients with only obsessional ruminations revealed significant reductions in the frequency of obsessions in 46% and reductions in distress in 12% of these patients [5]. Additionally, experimental studies on thought suppression suggested that attempts at suppression (including thought-stopping) could lead to an increased frequency of maladaptive thoughts [2]. Lastly, the treatment appears to lack a proper theoretical rationale [6]. However, it has been proposed that thought-stopping could be an effective treatment for OCD if it is used as a part of exposure and response prevention (ERP) and not as an independent treatment modality [1,2]. The combination of thought-stopping and ERP could be particularly useful in those with predominant obsessions without overt compulsions. These patients constitute about 20% of those with OCD and are poorly responsive to conventional ERP treatment [7]. Therefore, modifications of the ERP procedure that include thought-stopping as a method of response prevention could be effective in these patients [1,2]. Although there are reports of the use of thought-stopping as a sole strategy to treat obsessions, the combination of thought-stopping with ERP has not been used earlier. The case of a female with predominant obsessions is presented to illustrate the usefulness of a treatment approach integrating thought-stopping with ERP.

## Case presentation

This 61-year-old retired school teacher living with her husband and son in a large city in North India attended our outpatient clinic in September 2017. The patient had started worrying about her menstrual periods becoming scanty and finally stopping over two months in early 2005. On the advice of a colleague, she started praying more than usual to relieve her anxiety. However, while praying, she started to develop thoughts and images of setting fire to photographs, idols of deities, and temples or other holy places. Over the next few years, the thoughts/images of setting fire to places of worship began to occur whenever she passed by them. By early 2016, she had thoughts/images of setting fire to the house while cooking and thoughts/images of having sex with gods. She tried coping with these thoughts by concealment, suppression, and distraction. When these failed, she took to praying for forgiveness, avoiding places of worship, and her usual religious practices. She blamed herself, felt low, and had other depressive symptoms. Her work at home and school suffered. After her retirement, she started spending 10-12 hours a day praying. A succession of faith healers, physicians, and psychiatrists was consulted, but treatment including combinations of antidepressants and benzodiazepines brought no relief. When she started saying that she had committed a grave sin and would be better off dead, the family was alarmed and brought her to our outpatient clinic. She was eventually admitted to our inpatient ward for three months (from January to March 2018). She had some anankastic traits pre-morbidly, but these had not caused any significant impairment or distress. She had been diagnosed with hypothyroidism in the year 2000 and was on thyroxine since then. Around the same time, she had developed doubts about contamination and not locking doors properly with washing and checking compulsions. However, these symptoms had subsided on their own in three months.

Assessment

The diagnosis made was OCD with predominant obsessions and a moderate depressive episode. The criteria for an anankastic personality disorder were not met on the International Personality Disorder Examination. Pre-treatment scores of different aspects of her obsessive-compulsive symptoms are shown in Table 1. A detailed behavioral analysis was followed by the construction of an ascending hierarchy of antecedent situations and distressing thoughts/images.

**Table 1:** Rating the progress of treatment on different scales 1: The Yale-Brown Obsessive-Compulsive Scale rating was for obsessions only and indicated moderate obsessive-compulsive disorder (OCD) 2: The Hamilton Depression Rating Scale score indicated moderately severe depression 3: The Responsibility Interpretations Questionnaire [1] evaluates neutralization strategies. The average score of beliefs in the intensity of 16 "high responsibility" interpretations was estimated on a 0%-100% scale 4: The Structured Interview on Neutralization [7] is probe questions for the identified six neutralization strategies (thought suppression, distraction, thinking through, visualization, avoidance, and praying). The efficacy of these strategies was rated on a scale of zero (not at all effective) to four (extremely effective). Mean efficacy ratings were low at baseline (0.83). The frequency of these strategies was rated on a scale of zero (not used at all) to four (used all the time). Average frequency was calculated for all the six strategies 5: Emotional and Behavioral Reactions to Intrusions Questionnaire [8] is used to rate distress associated with intrusive thoughts. This seven-item, five-point rating scale ("never" to "every time") has a maximum score of 28

Scales	Pre-treatment scores	Scores after the first month	Scores after the second month	Scores after the third month (post-treatment)
Yale-Brown Obsessive-Compulsive Scale^1^	16	10	7	4
Hamilton Depression Rating Scale^2^	20	10	8	7
Responsibility Interpretations Questionnaire^3^	60%	45%	22%	14%
Structured Interview on Neutralization (frequency)^4^	16	13	8	3
Emotional and Behavioral Reactions to Intrusions Questionnaire^5^	26	20	12	4

Treatment

The treatment protocol is described in Table 2.

**Table 2:** The protocol for thought-stopping as a part of exposure and response prevention (ERP) treatment

Steps	Procedures
Behavioral analysis	Apart from identifying antecedents, consequences (neutralizations), and maintaining factors for obsessions, an ascending hierarchy of situations and obsessional thoughts was constructed, which formed the basis of treatment with thought-stopping and ERP.
Psychoeducation	The patient and her husband were educated about obsessive-compulsive disorder, as well as the behavioral treatment proposed, including the technique of thought-stopping.
Monitoring of progress	Both the patient and her husband were educated to monitor the frequency, associated distress, and impairment with obsessional symptoms. The therapist reviewed these ratings and also carried out structured ratings of his own.
Relaxation exercises	Autogenic training was taught according to a standardized protocol.
ERP and thought-stopping	The patient was exposed to the antecedent situations that brought on her obsessional symptoms according to the ascending hierarchy of her thoughts/images.
	The initial exposure sessions were carried out by presenting audio or video images of places of worship, fires, lighters, and matchsticks using a smartphone (imaginal exposure). She was taught thought-stopping during this period.
	Once she learned to use thought-stopping to successfully counteract the obsessions brought on by these situations, she was gradually exposed to real-life situations in a carefully graded manner.
Steps of thought-stopping	The patient was asked to lie down comfortably and do the relaxation exercises for 5-10 minutes. Anxiety levels were noted.
	The patient was asked to think about neutral topics that did not provoke anxiety.
	The antecedents to her obsessional thoughts/images were presented in the form of pictures and audio or video recordings.
	Whenever the obsessive thoughts/images interrupted her normal chain of thoughts, she indicated this by raising her finger.
	Once the patient signaled a disturbing thought/image, a loud sound was made by striking metal upon metal. The objective was to induce a startle reaction that would disrupt the patient's chain of thoughts/images. It was confirmed from the patient that the sound was not too unpleasant but loud enough to interrupt the chain of her thoughts/images.
	Once confirmed, the sound was repeated every time the patient indicated a disturbing thought/image for the entire session of about 30-45 minutes.
	A frequency count of the obsessive thoughts/images was done at the end of each session. Anxiety levels were noted again.
	Sessions for a particular antecedent situation and accompanying thoughts/images were repeated until the frequency of thoughts and anxiety levels decreased substantially over two to three consecutive sessions.
	The metal-on-metal sound was gradually replaced by metal-on-non-metal sound, non-metal-on-non-metal sound, both the patient and the therapist shouting stop, only the patient shouting stop, the patient saying stop in a normal voice, the patient saying stop under her breath (sub-vocally), the patient saying stop sub-vocally while flicking an elastic band on the wrist, and the patient "saying" stop in her "mind."
	The eventual aim was for the patient to control obsessive thoughts/images by "uttering" stop in her mind or under their breath. She could also use the rubber band discreetly.
Processing	After each session, the patient's understanding of the procedure was explored. During these sessions, she and her husband were educated about the maladaptive nature of obsessional thoughts. Neutralizing acts were pointed out, and the patient was asked to refrain from these. Instead, she was encouraged to use thought-stopping or other adaptive strategies to cope with obsessions during the sessions and between them.

The ERP treatment consisted of psychoeducation, monitoring, relaxation exercises, exposure sessions, and processing [9]. Processing involved discussing the patient's experience and understanding of the treatment after each session and how this met her expectations. Processing also allowed for discussions on the reality of the patient's beliefs, neutralization strategies, and the use of more adaptive strategies to cope with her thoughts/images. The techniques used to teach thought-stopping were based on previous reports [4,10-13]. Some of the steps such as relaxation exercises, asking the patient to think about neutral instead of distressing thoughts, and graded exposure according to an ascending hierarchy had been used earlier [10-13]. Unlike these studies, both imaginal and live exposures were used in our patient. For example, the patient was first exposed to photographs of temples on a smartphone, followed by a large screen, and finally, she was asked to visit temples in a graduated manner. The patient was encouraged to use thought-stopping to counteract anxiety and avoid using neutralization strategies during sessions, as well as between the sessions. She underwent two sessions daily, one supervised by the therapist and the other by her husband (a total of 70 sessions). She also received sertraline 150 mg/day during this period.

The progress of therapy according to the scores on different rating scales is depicted in Table 1. The patient improved in all aspects including obsessions, depressive symptoms, intrusiveness, the distress accompanying obsessions, and the frequency of neutralizing acts. Both the patient and her husband who were initially skeptical about the behavioral treatment were satisfied with the outcome and agreed to continue the treatment at home. She suffered a relapse in the first few months after discharge but responded to videoconferencing-based treatment sessions supervised by the therapist and aided by her husband. She was free of symptoms and functioned adequately for over two years following this relapse.

## Discussion

Thought-stopping is no longer used to treat OCD because of concerns about its ineffectiveness, the detrimental effects of thought suppression, and the uncertainty about its underlying mechanisms [1-5]. Our patient had religious obsessions, which are more common in non-Western cultures and more likely to be associated with anxiety, depression, and guilt [14]. However, instead of overt compulsions, she was using neutralization strategies to reduce distress, which became the principal cause of her subsequent impairment. Clark [1] has suggested that thought-stopping as a sole treatment may be useful in patients with distressing thoughts but no overt compulsions. When thought-stopping is taught systematically, the patient acquires a cognitive control technique that disrupts obsessional thoughts and the link between these thoughts and neutralization strategies [2,15]. In contrast, thought suppression is a neutralizing strategy commonly used by patients in a non-systematic fashion. It provides only temporary relief from obsessions. However, the use of thought-stopping as a sole technique may be only partially effective or completely ineffective in many patients [1-4]. Therefore, the key to using thought-stopping effectively in OCD is to make it a part of an ERP protocol. The combination not only disrupts obsessional thinking but also prevents neutralization (which is equivalent to overt compulsions) from occurring [2]. The cognitive model of OCD suggests that a two-step procedure can be effective in patients with predominant obsessions [5,6].

Exposure to anxiety-arousing situations generates distressing thoughts/images and the urge to carry out neutralization strategies. With repeated exposure, habituation can increase the patient's self-control and ability to resist their familiar neutralizing acts leading to a reduction of such urges [15]. Therefore, when thought-stopping follows exposure, it acts as a means of response prevention for blocking the neutralizations that prevent habituation [16]. This was probably the mechanism underlying the effectiveness of thought-stopping integrated with ERP in this patient with predominant obsessions. The alternative to thought-stopping as a part of this combined treatment is cognitive restructuring, which is also an effective strategy to prevent neutralization [7]. ERP and cognitive restructuring have been successfully used earlier to treat patients with predominant obsessions without overt compulsions in a trial [17]. We attempted cognitive restructuring but failed because she (and her husband) had difficulty in understanding and accepting what appeared to be an unfamiliar technique for them. Instead, the discussions about the reality of her beliefs and the ineffectiveness of neutralization strategies during processing after the exposure sessions proved more helpful. Although ERP is primarily a behavioral procedure, it can reduce dysfunctional cognitions by providing corrective information. Thus, discussions about maladaptive cognitions during the ERP treatment can reduce the frequency and intensity of obsessions while encouraging the patient to refrain from indulging in neutralizing acts [18].

All the earlier reports of thought-stopping in the treatment of predominant obsessions have used it as a sole treatment strategy [9-13]. Some of these studies have also used graded exposure as a part of thought-stopping [9,11]. However, the use of thought-stopping as a response prevention measure to prevent neutralization together with processing to address dysfunctional cognitions has not been tried earlier. Even so, the improvement in this patient cannot be immediately ascribed to these techniques because other mechanisms of change such as escape learning, self-monitoring, stimulus control, and non-specific factors may also be involved [2-6].

## Conclusions

Although the combination of thought-stopping, exposure, and simple techniques to address dysfunctional cognitions appeared to have worked in this patient, conclusions based on the treatment of a single patient are difficult. Then again, she had chronic and progressively worsening OCD that had not responded to multiple antidepressants. Therefore, her eventual improvement cannot be solely ascribed to medications, spontaneous remission, or any other interventions apart from the combination of thought-stopping and ERP. The treatment approach of combining thought-stopping with ERP in this patient differed from the earlier studies that had used thought-stopping as an independent modality to treat OCD. Therefore, the successful treatment of OCD in this patient suggests that it might be worthwhile to examine the effectiveness of combining thought-stopping with ERP in properly controlled trials of patients with predominant obsessions.
